# The multifunctional FUS, EWS and TAF15 proto-oncoproteins show cell type-specific expression patterns and involvement in cell spreading and stress response

**DOI:** 10.1186/1471-2121-9-37

**Published:** 2008-07-11

**Authors:** Mattias K Andersson, Anders Ståhlberg, Yvonne Arvidsson, Anita Olofsson, Henrik Semb, Göran Stenman, Ola Nilsson, Pierre Åman

**Affiliations:** 1 Lundberg Laboratory for Cancer Research, Department of Pathology, Sahlgrenska Academy at Göteborg University, Göteborg, Sweden; 2 Center for Brain Repair and Rehabilitation (CBR), Department of Clinical Neuroscience and Rehabilitation, Sahlgrenska Academy at Göteborg University, Göteborg, Sweden; 3 Stem Cell Center, Lund University, Lund, Sweden

## Abstract

**Background:**

FUS, EWS and TAF15 are structurally similar multifunctional proteins that were first discovered upon characterization of fusion oncogenes in human sarcomas and leukemias. The proteins belong to the FET (previously TET) family of RNA-binding proteins and are implicated in central cellular processes such as regulation of gene expression, maintenance of genomic integrity and mRNA/microRNA processing. In the present study, we investigated the expression and cellular localization of FET proteins in multiple human tissues and cell types.

**Results:**

FUS, EWS and TAF15 were expressed in both distinct and overlapping patterns in human tissues. The three proteins showed almost ubiquitous nuclear expression and FUS and TAF15 were in addition present in the cytoplasm of most cell types. Cytoplasmic EWS was more rarely detected and seen mainly in secretory cell types. Furthermore, FET expression was downregulated in differentiating human embryonic stem cells, during induced differentiation of neuroblastoma cells and absent in terminally differentiated melanocytes and cardiac muscle cells. The FET proteins were targeted to stress granules induced by heat shock and oxidative stress and FUS required its RNA-binding domain for this translocation. Furthermore, FUS and TAF15 were detected in spreading initiation centers of adhering cells.

**Conclusion:**

Our results point to cell-specific expression patterns and functions of the FET proteins rather than the housekeeping roles inferred from earlier studies. The localization of FET proteins to stress granules suggests activities in translational regulation during stress conditions. Roles in central processes such as stress response, translational control and adhesion may explain the FET proteins frequent involvement in human cancer.

## Background

Gene expression was for a long time considered to consist of a chain of distinct events starting with synthesis of RNA, followed by splicing and ending with mature mRNAs being translated in the cytoplasm. The discovery of multifunctional RNA-binding proteins has since then joined transcription, RNA processing, transport of RNA species and translation into an integrated tightly regulated cellular machinery [1,2]. One such group of proteins is the FET (previously called TET) family of RNA-binding proteins [3]. The FET family consists of mammalian FUS (TLS) [4], EWS [5], TAF15 (TAFII68, TAF2N, RBP56) [3] and the closely related Drosophila cabeza/SARFH [6]. All four proteins are structurally similar and contain a number of evolutionary conserved regions [7]. The FUS, EWS and TAF15 proteins bind RNA as well as DNA and have both unique and overlapping functions. The human FET proteins are associated with transcription, splicing, microRNA (miRNA) processing [8,9], RNA transport, signaling and maintenance of genomic integrity. Furthermore, the 5' parts of the human FET genes are as a result of chromosomal translocations rearranged and fused to various transcription factor genes in multiple human malignancies. These events are considered the driving forces of cancer development in their associated diseases [2,10].

Although the FET family proteins are implicated in numerous cellular processes their functions remain poorly characterized. This together with the fact that the proteins are structurally similar prompted us to investigate their cell type-specific expression. In the present study, we used immunostaining and ectopically expressed proteins to examine the expression patterns of FET family members in multiple human tissues and cell types. Our results show that the three FET proteins are heterogeneously expressed throughout human tissues with FUS and TAF15 having highly correlated expression patterns. In addition, we here report that the FET proteins display alterations in expression at both mRNA and protein level upon differentiation and that they are involved in cellular stress response as well as cell spreading.

## Results

### FUS, EWS and TAF15 show cell type-specific localization in vivo

**Figure 1:**
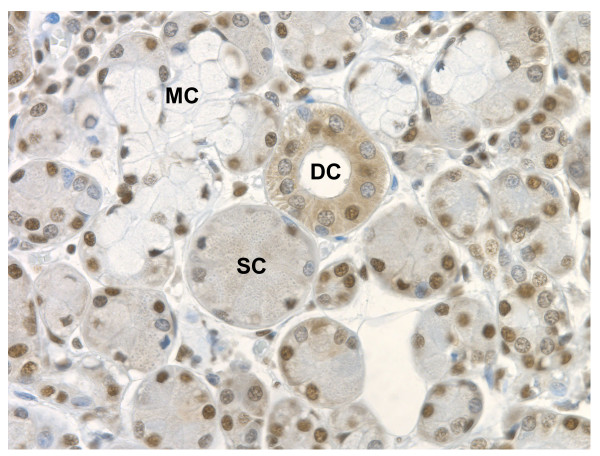
**EWS expression in salivary gland**. EWS shows cytoplasmic expression in ductal and serous cells but is undetectable in the cytoplasm of mucous cells. DC – ductal cells, MC – mucous cells, SC – serous cells.

Tissue microarrays (TMA) were stained with antibodies against FUS, EWS and TAF15 and the percentage of positively staining cells within 35 organs were estimated (Table 1). The FET proteins showed almost ubiquitous expression with FUS and TAF15 having highly correlated expression patterns (Table 2). However, FET proteins were not detected in melanocytes and cardiac muscle cells and neither FUS nor TAF15 were detected in cardiac endothelium. Most cell types showing FET expression had nuclear localization of the proteins but FUS and TAF15 was absent from the nuclei of hepatocytes. Moreover, FUS and TAF15 often showed cytoplasmic localization while EWS was more rarely found in this compartment (Table 1). Cytoplasmic EWS was mainly detected in secretory cell types. In salivary gland, EWS showed a striking divergence in cytoplasmic expression between different cell types. EWS was restricted to the nuclei of mucous cells while being expressed in both the nucleus and cytoplasm of ducts and serous cells (Figure 1).

**Table 1:** Expression patterns of FUS, EWS and TAF15 in human tissues by TMA immunohistochemistry analysis

		**FUS**		**EWS**		**TAF15**	
**Organ**	**Tissue/Cell type**	Nuclear	Cytoplasmic	Nuclear	Cytoplasmic	Nuclear	Cytoplasmic
Adrenal gland	Parenchymal cells	+++	+++	+++	-	+++	+++
Appendix	Appendix smooth muscle	+++	+++	+++	-	+++	+++
Brain							
- white matter	Glial cells	+++	-	+++	-	+++	-
- grey matter	Neurons	+++	+	+++	-	+++	+
Breast	Glandular epithelium	+++	+++	+++	-	+++	+++
	Myoepithelial cells	+++	+++	++	-	+++	+++
	Endothelium, capillaries	+++	++	+++	-	+++	+++
	Adipocytes	+++	-	+++	-	+++	-
Colon & Rectum	Surface epithelium	+++	+++	++	-	+++	+++
	Crypt epithelium	+++	+++	++	-	+++	+++
	Malt tissue, germinal centre	+++	-	+++	-	+++	-
	Malt tissue, mantle zone	+++	-	+++	-	++	-
Duodenum	Enterocytes, villus epithelium	+++	+++	++	-	+++	+++
	Enterocytes, crypt epithelium	+++	+++	++	-	+++	+++
	Ganglion cells	+++	+++	+++	-	+++	+++
Endometrium							
- proliferative	Glandular epithelium	+++	+++	+++	-	+++	+++
	Stromal cells	+++	-	++	-	+++	-
- secretory	Glandular epithelium	+++	+++	+++	-	+++	+++
	Stromal cells	++	-	++	-	++	-
Esophagus	Squamous epithelium, suprabasal	+++	+++	+++	-	+++	+++
	Squamous epithelium, basal	++	+++	+	-	++	+++
	Endothelium, medium-sized arteries	+++	+	+++	-	+++	++
	Smooth muscle cells	+++	+++	+++	-	+++	+++
Gallbladder	Mucosal epithelium	+++	+++	+++	+++	+++	+++
	Smooth muscle cells	+++	+++	+++	-	+++	+++
Heart	Cardiac muscle cells	-	-	-	-	-	-
	Cardiac endothelium	-	-	++	-	-	-
Kidney	Glomeruli	++	-	++	-	++	-
	Proximal tubuli	+++	+++	+++	+++	+++	+++
	Distal tubuli	+++	+++	+++	+++	+++	+++
Liver	Hepatocytes	-	+++	++	+++	-	+++
	Bile duct epithelium	+++	+++	+++	+++	+++	+++
Lung & Bronchus	Respiratory epithelium	+++	+++	+++	-	+++	+++
	Glandular epithelium	+++	+++	+++	-	+++	+++
	Pneumocytes	++	-	++	-	++	+
	Chondrocytes	+++	-	++	-	++	-
	Endothelium, capillaries	+++	-	+++	-	+++	-
	Muscle artery, endothelial cells	+++	-	+++	-	+++	-
	Muscle artery, smooth muscle	+++	+++	+++	-	+++	+++
Lymph node	Germinal centre	++	-	++	-	++	-
	Mantle zone	++	-	++	-	++	-
Myometrium	Smooth muscle cells	+	-	+	-	+	-
Nasal mucosa	Respiratory epithelium	+++	++	++	-	+++	++
	Glandular epithelium	+++	+++	+++	-	+++	+++
Ovary	Follicular epithelium	+++	+++	++	-	+++	+++
	Ovarial stromal cells	+++	-	+++	-	+++	-
Pancreas	Exocrine glands	+++	+++	+++	-	+++	+++
	Duct epithelium	+++	+++	+++	-	+++	+++
	Endocrine cells	+++	+++	+++	+++	+++	+++
Placenta	Syncytiotrophoblasts	++	++	+	+	++	+++
	Cytotrophoblasts	+++	+	++	+	+++	+
Prostate	Prostate epithelium	+	+	++	+	++	+
	Basal cells	+++	-	+++	-	++	-
Salivary gland	Serous cells	+++	+++	+++	++	+++	+++
	Mucous cells	+++	+	++	-	+++	+
	Intercalated/striated ducts	+++	+++	+++	+++	+++	+++
	Myoepithelium	+++	-	+++	-	+++	-
Salpinx	Glandular epithelium	+++	+++	+++	-	+++	+++
Seminal vesicle	Epithelium	+++	+++	+++	+++	+++	+++
Skeletal muscle	Skeletal muscle fibers	++	-	++	-	+++	-
Skin & Subcutis	Keratinocytes	+++	+++	+++	-	+++	+++
	Melanocytes	-	-	-	-	-	-
	Fibroblasts	+++	-	+++	-	+++	-
	Ecrine epithelium, sweat gland	+++	+++	++	-	+++	+++
	Endothelium, capillaries	+++	-	+++	-	+++	-
	Adipocytes	+++	-	++	-	++	-
Spleen	Lymphocytes, red and white pulp	++	-	++	-	+	-
Stomach							
- body	Surface & foveolar epithelium	+++	+++	+++	-	+++	+++
	Specialized glandular epithelium	+	+++	+	+++	+	+++
- antrum	Surface & foveolar epithelium	+++	+++	++	-	+++	+++
	Specialized glandular epithelium	+++	+++	++	-	+++	++
- smooth muscle	Smooth muscle cells	+++	-	+++	-	+++	-
Small bowel	Enterocytes, villus epithelium	+++	+++	++	-	+++	+++
	Enterocytes, crypt epithelium	+++	+++	++	-	+++	+++
	Ganglion cells	+++	+++	+++	+++	+++	+++
Testis	Germinal epithelium	+++	-	+++	-	+++	-
	Leydig intestitial cells	++	++	++	-	++	+++
	Spermatides	+	-	+	-	++	-
Thymus	T-lymphocytes	++	-	++	-	++	-
	Epithelium	+++	+	+++	-	+++	+++
Thyroid	Follicular epithelium	+++	+++	++	-	+++	+++
Tonsil	Squamous cell epithelium	+++	+++	+++	-	+++	+++
	Lymphocytes	+++	-	+++	-	+++	-
Umbilical cord	Stromal cells	+++	++	++	-	+++	++
Urinary bladder	Urothelium	+++	+++	+++	-	+++	+++
Uterine cervix	Squamous epithelium, basal	+++	+++	++	-	+++	+++
	Squamous epithelium, suprabasal	+++	+++	+++	-	+++	+++

The percentage of FET positive cells within an organ is indicated as: +++ (100-76% positively staining cells), ++ (75-26% positively staining cells), + (25-1% positively staining cells) and - (negative).

**Table 2:** Calculated Spearman correlations of FET expression patterns from Table 1

	**FUS **N	**FUS **CP	**EWS **N	**EWS **CP	**TAF15 **N	**TAF15 **CP
**FUS **N	1	0.40**	0.67**	-0.08	0.87**	0.35**
**FUS **CP		1	0.19	0.29**	0.47**	0.96**
**EWS **N			1	0.06	0.63**	0.19
**EWS **CP				1	-0.04	0.30**
**TAF15 **N					1	0.43**
**TAF15 **CP						1

Asterisks indicate statistical significance with p < 0.01. N – nuclear, CP – cytoplasmic.

### The FET proteins show conserved localization in cultured cells

**Figure 2:**
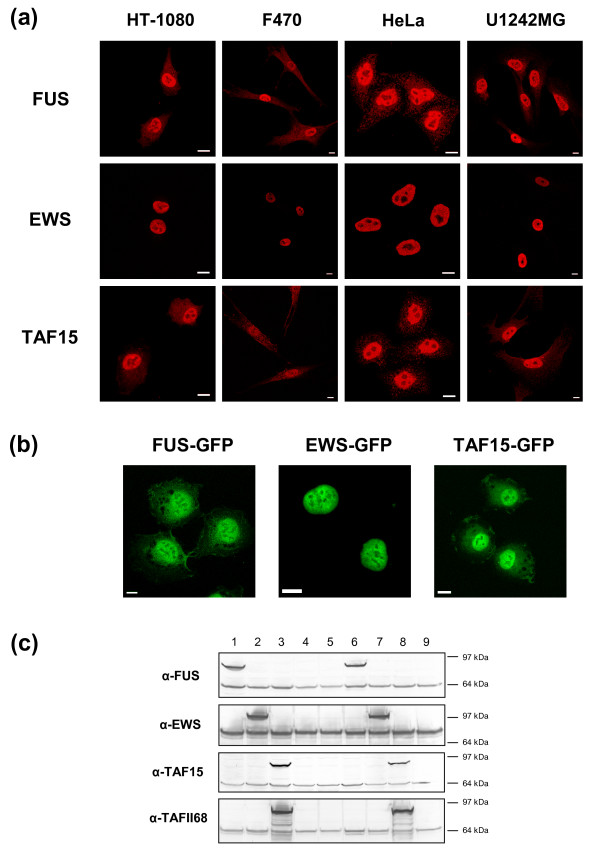
**FET protein localization in cultured human cells**. **(a) **Actively proliferating cells stained with antibodies for FET proteins. FUS and TAF15 show both nuclear and cytoplasmic localization while EWS is found solely in nuclei. Scale bars indicate 10 μm. **(b) **HT-1080 cells stably expressing FET-GFP proteins show nuclear localization of all three proteins and in addition cytoplasmic localization for FUS-GFP and TAF15-GFP. Scale bars indicate 10 μm. **(c) **Western blots showing FET-GFP proteins of expected sizes and specificity of FET antibodies used. Wells contain the following lysates: FUS-GFP clone 1 (1), EWS-GFP clone 1 (2), TAF15-GFP clone 1 (3), GFP clone 1 (4), HT-1080 (5), FUS-GFP clone 2 (6), EWS-GFP clone 2 (7), TAF15-GFP clone 2 (8), GFP clone 2 (9). No crossreactivity is seen between different FET antibodies. Endogenous FET proteins correspond to the lower bands seen in all lanes and tagged FET proteins to upper bands. GFP adds approximately 27 kDa to the total size of the respective protein. FUS-GFP and EWS-GFP are expressed at slightly augmented levels compared with their endogenous counterparts while TAF15-GFP is expressed at much higher levels than wild type TAF15.

In cultured human cells, the FET proteins displayed smooth nuclear localization with infrequent nuclear speckles (Figure 2a and Additional file 1). None of the proteins localized to nucleoli. FUS and TAF15 showed a diffuse distribution in the cytoplasm but were in some cases localized to small cytoplasmic granules. EWS was not detected in the cytoplasm. These patterns were similar for all cell lines tested. Stably expressed GFP-tagged FET proteins demonstrated similar expression patterns as the corresponding endogenous proteins (Figure 2b) while overexpressed FET proteins showed protein aggregation (see below). FET protein expression in stable transfectants was confirmed by western blot analysis (Figure 2c). Cell measurements performed on stable transfectants showed that cells with ectopic FUS or FUSA expression were somewhat larger than other cells (Additional File 2).

### The FET family proteins are targeted to stress granules upon environmental stress

**Figure 3:**
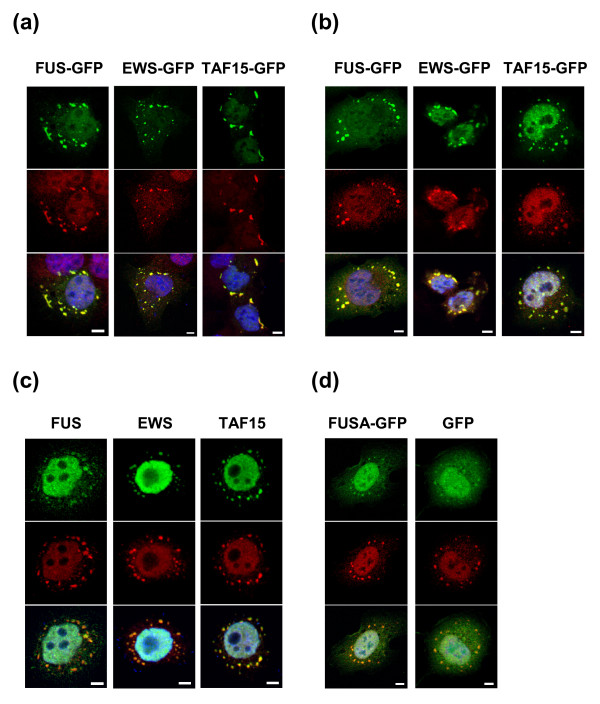
**FET proteins localize to stress granules**. Images show FET proteins (green) and immunostaining for the stress granule marker TIA-1 (red). Nuclei are visualized by DAPI staining (blue) in the bottom image of each column **(a) **Overexpression of FET-GFP proteins cause stress granule formation in transiently transfected HT-1080 cells. **(b) **HT-1080 cells stably expressing FET-GFP proteins show localization of tagged proteins to stress granules upon arsenite treatment. **(c) **Endogenous FET proteins localize to stress granules in response to oxidative stress in HeLa cells. **(d) **Stable transfectants of FUSA-GFP and GFP show similar minor signal in granules upon arsenite treatment. Scale bars indicate 5 μm.

Cells transiently transfected with FET-GFP expression vectors showed occasional nuclear and more commonly cytoplasmic FET-GFP protein aggregation (Figure 3a). The cytoplasmic aggregates resembled stress granules (SGs) [11]. When such cells were stained with antibodies against the SG marker TIA-1 [12], colocalization was seen between this marker and the ectopically expressed GFP-tagged FET proteins (Figure 3a). However, there were clear differences between the individual FET proteins. EWS-GFP rarely localized to SGs (in less than 1% of the cells), while FUS-GFP and TAF15-GFP localized to stress granules in the majority of transiently transfected cells. To further confirm that the FET proteins localize to SGs following stress, stable FET transfectants (not showing stress granules under normal growth conditions (Figure 2b)) and HeLa cells were exposed to oxidative stress by sodium arsenite treatment (Figure 3b and 3c). FET-GFP proteins as well as endogenous FET proteins localized to stress granules following these experiments. EWS-GFP was only detected in a minority of cells and in cases were two smaller cells were located in close proximity to each other. Endogenous EWS on the other hand was found in SGs in all HeLa cells. The FET proteins showed similar SG localization after both heat-shock and arsenite treatment (not shown). A stably expressed FUS mutant lacking the RNA-binding domain showed background signal in SGs similar to the GFP control (Figure 3d).

### FUS and TAF15 localize to spreading initiation centers

**Figure 4:**
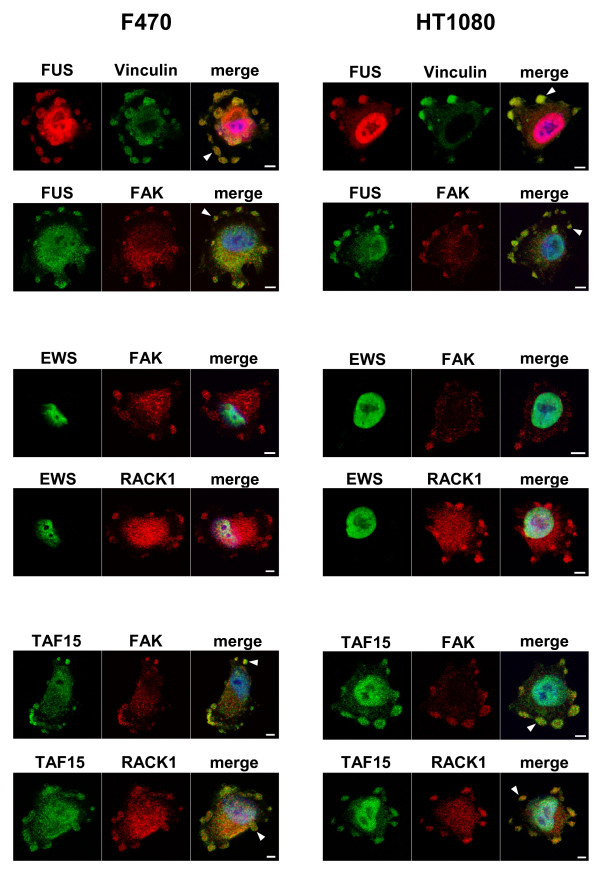
**FUS and TAF15 localize to spreading initiation centers**. F470 and HT-1080 cells co-stained with FET antibodies and focal adhesion/SIC markers Vinculin, FAK and RACK1. Arrowheads indicate areas of cytoplasmic overlap and nuclei are shown in blue by DAPI staining in the merge images. Bars indicate 5 μm.

FUS and TAF15, but not EWS, were detected in large cytoplasmic granules forming close to the plasma membrane in a subset of adhering F470 and HT-1080 cells. These cells were rounded-up and seemingly not completely attached to the underlying surface. When such cells were further co-stained with the focal adhesion/spreading initiation center (SIC) markers Vinculin, FAK and RACK1 [13], colocalization was apparent between these markers and both FUS and TAF15 (Figure 4).

### FUS, EWS and TAF15 show heterogeneous expression within the same cell type *in vivo*

**Figure 5:**
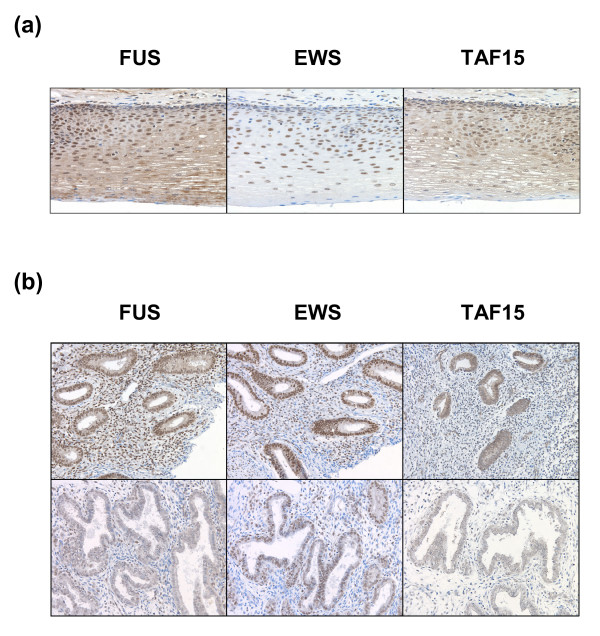
**Heterogenous FET protein expression in human tissues**. Brown staining indicates FET expression while blue staining shows negatively staining nuclei. **(a) **Individual epithelial cells of esophagus show large heterogeneity in FET expression levels. **(b) **FET expression is elevated in proliferating endometrium (upper panel) compared to differentiated secretory endometrium (lower panel).

When comparing individual cells of the same type within tissues, we found that in addition to differences in localizations there were quantitative differences in the amount of FET protein expression. Epithelial cells of the esophagus displayed the most pronounced variations ranging from strong to complete lack of expression within the same cell type (Figure 5a). Similar observations were made for many other cell types and tissues (not shown). In addition, markedly attenuated FET expression was seen in secretory endometrium compared with proliferative endometrium (Figure 5b). Immunostained cultured cells showed more homogeneous FET expression (Figure 2a).

### FET family expression is downregulated in neuroblastoma cells following retinoic acid treatment

**Figure 6:**
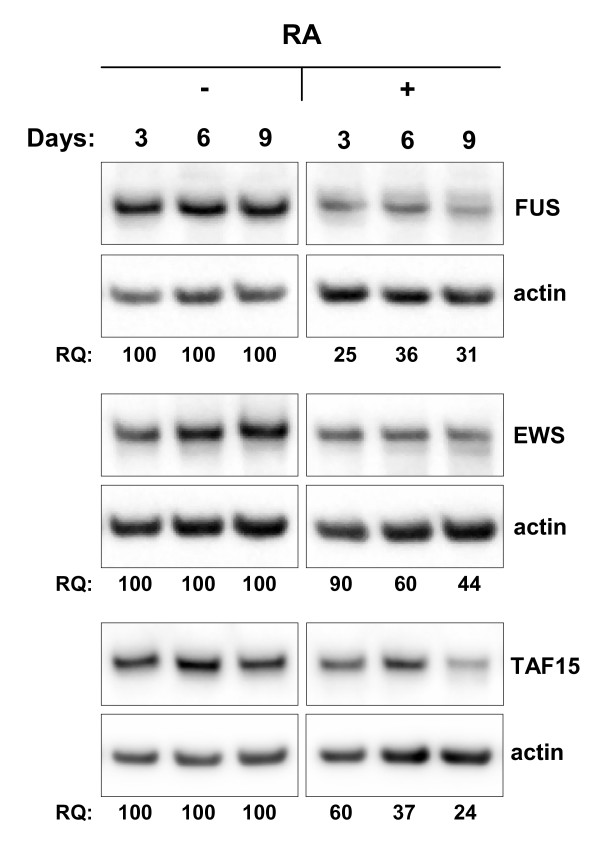
**FET expression is reduced during neuronal differentiation of SH-SY5Y neuroblastoma cells**. Cells were treated with 1 μM of all-trans retinoic acid and lysed at different time points. Relative quantification (RQ) values were obtained by normalizing FET expression against beta actin expression in each sample and by further comparing RA treated with untreated cells at each time point. Data from one of two independent experiments yielding similar results.

To investigate a possible connection between FET expression and differentiation, we analyzed FET expression in SH-SY5Y neuroblastoma cells treated with all-trans retinoic acid (RA). Cells were monitored by light microscopy and harvested after 3, 6 and 9 days of treatment. An increased neurite formation was visual in RA treated cells compared to untreated cells. Western blot analysis of protein extracts from these cells demonstrated a marked decrease in FET expression upon prolonged retinoic acid treatment (Figure 6). No change in localization of the FET proteins was detected by immunofluorescence analysis after RA treatment of SH-SY5Y cells (not shown).

### FUS, EWS and TAF15 gene expression is gradually attenuated in differentiating human embryonic stem cells

**Figure 7:**
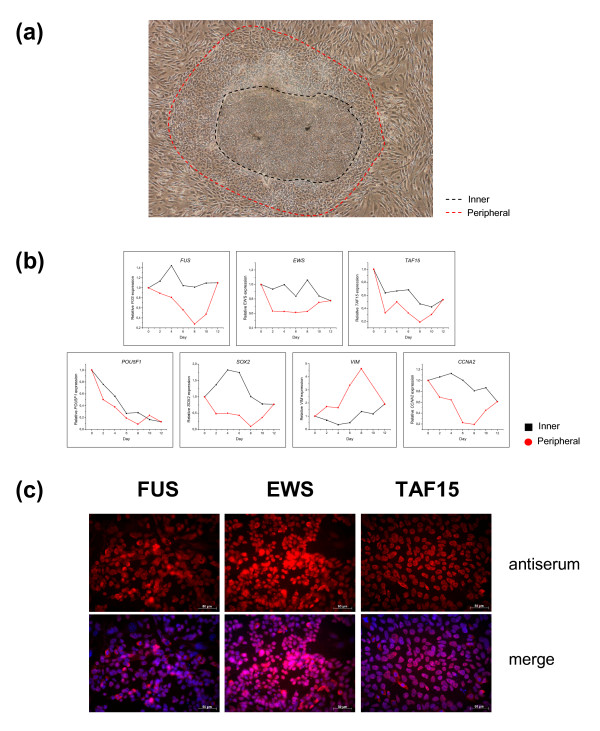
**FET gene expression is attenuated in differentiating human embryonic stem cells**. **(a) **A colony of spontaneously differentiating hESCs. Inner and peripheral parts of the colonies were harvested based on cell morphology. **(b) **Relative expression of selected mRNAs in samples from inner or peripheral parts of colonies analyzed by quantitative real-time PCR with *ACTB *as endogenous control. *POU5F1 *– pluripotency marker,*SOX2 *– ectodermal marker, *VIM *– mesodermal marker,*CCNA2 *– proliferation marker. The day 12 measurement consists of one sample taken from inseparable, mixed cell populations. **(c) **Undifferentiated hESCs show positive FET protein staining. Cells were stained with primary antibodies for FET proteins and visualized with Cy3-conjugated secondary antibodies (red). The merge images additionally show DAPI staining of nuclei (blue).

We further investigated FET family gene expression during spontaneous differentiation of cultured human embryonic stem cells (hESCs). Samples were collected from morphologically distinct inner and peripheral parts of the hESC colonies (Figure 7a). Quantitative RT-PCR was used to assay FET gene expression as well as markers for differentiation and proliferation (Figure 7b). FET gene expression was over time downregulated in the peripheral parts of the hESC colonies. However, the expression of individual FET genes diverged partially from each other. *FUS *and *TAF15 *expression was considerably reduced in the peripheral parts while *EWS *showed only weak attenuation here. In addition, *TAF15 *was slightly downregulated in the inner parts, while *FUS *and *EWS *expression was maintained in these cells. An overall downregulation of *POU5F1 *(*OCT4*) indicated that all hESCs had initiated differentiation (*POU5F1 *expression is restricted to undifferentiated cells). Over time, peripheral colonies spontaneously differentiated towards a mesodermal cell fate defined by an upregulation of *VIM *expression. The inner parts showed a very weak upregulation of *SOX2*, an early ectodermal marker as well as downregulation of the proliferation marker *CCNA2*. FUS, EWS and TAF15 expression at the protein level was confirmed in hESCs by immunofluorescence (Figure 7c).

### FET protein expression is maintained in growth arrested cells and unaltered by serum starvation or stimulation

As FET gene expression correlated with the proliferation marker *CCNA2 *in hESCs we investigated whether actively proliferating cells have increased FET expression compared to quiescent cells. Proliferating, semi-confluent F470 primary human fibroblasts were compared with confluent contact-inhibited cells. Also, experiments studying FET expression following serum-stimulation/starvation were performed in HT-1080 cells. These experiments did no reveal any significant differences in FET expression as analyzed by western blot (Additional file 3).

## Discussion

In the present study, we show that the FET proteins are ubiquitously expressed throughout human tissues and only a few cell types lack FET expression. The proteins display cell type-specific localization with FUS and TAF15 having highly similar expression patterns. FUS and EWS have previously been found in the nucleus as well as in the cytoplasm and shown to shuttle between these locations [14,15]. We here report that TAF15 is also present in both of these compartments in numerous human cell types. This implies that TAF15 may participate in nuclear-cytoplasmic shuttling in much the same way as the other FET family members do. In general, FUS and TAF15 showed cytoplasmic localization in most cell types while EWS was more rarely seen in this compartment. Expression data obtained for EWS in the present study differed from that recently made available on the internet [16], where the EWS expression was judged to be restricted to the nucleus throughout human tissues. Differences in staining procedure and analysis could explain discrepancies between our studies. In this study, cytoplasmic EWS was mainly detected in secretory cell types, suggesting that EWS is involved in the expression of secreted proteins. In salivary gland, the EWS protein was expressed in both the cytoplasm and nucleus of ductal and serous cells. In contrast, EWS was restricted to a nuclear localization in mucous cells. This and other examples of differential expression of the FET proteins in closely related cell types indicate specific roles in regulation of specialized functions. In cultured cells of different tissue origins, EWS expression was limited to the nucleus whereas FUS and TAF15 were observed in both compartments. This pattern reflected the most common type of expression seen in tissues and may be explained by homogeneous culture conditions. The strong nuclear preference of EWS in most cell types in contrast to FUS and TAF15 may be connected to its more frequent mutation in human cancers were the oncoprotein is restricted to nuclear functions as an aberrant transcription factor. The localization of normal EWS in various subcellular compartments has previously been shown to be affected by methylation [17] but it is unknown whether localization of FUS and TAF15 is regulated in a similar manner.

A majority of cells overexpressing GFP-tagged FUS and TAF15 showed cytoplasmic aggregates that colocalized with the stress granule marker TIA-1. These results implied that the FET proteins might target SGs as part of cellular stress response. SGs are phase-dense particles that are composed of stalled translation pre-initiation complexes, mRNAs and RNA-binding proteins and appear in the cytoplasm of cells exposed to environmental stress [11]. To further confirm SG localization of FET proteins, we exposed stable FET transfectants and HeLa cells to oxidative stress and heat shock. Exogenous as well as endogenous FET proteins localized to SGs in these experiments. However, exogenous EWS was detected in SGs only in occasional cells and endogenous EWS showed weak SG staining compared to FUS and TAF15. This could be explained by higher amounts of cytoplasmic EWS during and post mitosis. FUS was recently reported in stress granules in a small subset of thapsigargin treated cells and also when expressed as an RFP-tagged protein [18]. We here further show that the entire FET family is targeted to SGs upon environmental stress. FUSA and GFP expressing cells showed similar degrees of signal in SGs which seemed to correlate with the amount of GFP tagged protein present in the cell. It is therefore possible that a certain amount of ectopically expressed protein associates with stress granules as a consequence of overexpression and non-specific protein aggregation. The SG signals from the full-length FET members were judged to far exceed those of the FUS mutant and the GFP protein alone. It is therefore likely that the RNA-binding domain of FUS is needed for stress granule targeting. Several other RNA-binding proteins have previously been found in stress granules, e.g. TIA-1, HuR, hnRNPA1, YB-1 and FMRP [19-21], and many of these proteins have been shown to regulate translation of specific mRNAs [22-25]. For the FMRP protein, this regulation was proposed to be mediated by miRNA-coupled translational repression [25]. FMRP, hnRNPA1 and YB-1 have previously been found together with FUS and EWS in messenger ribonucleoprotein complexes [26,27], suggesting related functions of these proteins. In addition, the FET proteins are part of nuclear miRNA processing complexes [8,9] and in our work detected in cytoplasmic SGs known to contain miRNAs [28]. Hence, it is possible that the FET proteins interact with miRNAs and shuttle in protein-RNA complexes containing these non-coding RNAs. FUS has previously been associated with polysomes [29] and implicated in regulation of localized protein synthesis in dendritic spines [30]. The protein is also proposed to be a component of processing bodies, cellular structures with a direct role in mRNA degradation and with implications in RNAi-mediated post-transcriptional gene silencing [21]. However, we could not detect any structures resembling p-bodies and the FET family was restricted to stress granules in our hands. Based on these data we speculate that the FET proteins have functions in regulation of post-transcriptional gene expression during both normal and stress-induced situations.

The FUS protein has earlier been detected in spreading initiation centers, focal adhesion-like complexes that assembles upon early cell spreading [13]. We here show that TAF15 is also present in these structures. However, EWS was undetectable in SICs, possibly is due to a low abundance of cytoplasmic EWS protein under normal conditions. FUS has previously been reported in NMDA receptor-adhesion protein signaling complexes [31] and all three FET proteins have been found to interact with v-Src [32], a protein known to indirectly induce adhesion turnover and actin remodeling [33]. de Hoog *et al*. have also found that perturbation of RNA-binding proteins (in particular FUS) affects cell spreading [13]. These data suggest that at least two of the FET proteins are involved in focal adhesion-related processes. We noted that stable transfectants expressing FUS variants were somewhat larger than other stably transfected cells but seemingly not flatter when visually inspected in the z-axis by confocal microscopy. The reason for this larger phenotype of FUS-expressing cells and a putative connection with cell spreading is currently not understood.

In addition to *in vivo *cell type-specific localization, the FET proteins showed heterogeneous expression levels within the same cell type in multiple organs. These results suggested that the expression of the FET proteins may be regulated in individual cells by external factors provided by neighboring cells and the microenvironment. Observations from cell cultures with uniform growth conditions and cell populations, showing only small variances in FET expression between individual cells, supported this assumption. However, we saw no change in FET expression following experimental serum starvation or stimulation of fibrosarcoma tumor cells, implying that externally provided serum factors are not directly affecting FET expression in these cells. An alternative hypothesis is that the observed heterogeneity depends on differentiation, attributing a developmental role for the proteins. We investigated this hypothesis experimentally by measuring FET expression in SH-SY5Y neuroblastoma cells induced to differentiate by retinoic acid. In these experiments, FET expression was markedly decreased in cells receiving RA treatment compared to untreated cells. To further investigate expression of the FET family during differentiation, we measured FET gene expression in spontaneously differentiating human embryonic stem cells. We conclude from these data that the three FET genes are regulated upon early differentiation in a lineage-specific manner.

Altered FUS and EWS expression during differentiation has been reported by several studies [34-37]. Furthermore, FUS and EWS have been shown to be required for B lymphocyte development and for spermatogenesis in mice [38-40]. One study showed that an alternative EWS isoform is augmented during neuronal development [41]. In the present study, we could however not distinguish between EWS isoforms. Nevertheless, our data together with previous reports point to functions for the FET family in specialized cells, rather than the housekeeping functions inferred from earlier promoter studies [42-44]. This conclusion is further supported by the lack of FET expression in terminally differentiated melanocytes and cardiac muscle cells. Bertolotti *et al*. previously showed that the FET proteins associate with both common and distinct TFIID complexes and RNA polymerase II subunits [3,45]. These results implied overlapping as well as unique functions within the FET family. Our data showing differences in regulation and expression patterns of the individual FET proteins in specific cell types supports this interpretation and suggests that these unique functions are manifested at a cell type-specific level.

A previous study showed that the FUS homolog pigpen is regulated during the transition between proliferating and quiescent endothelial cells [46], providing an alternative explanation for the heterogenic FET expression seen in tissues and under experimental conditions. Therefore, we investigated a possible correlation between proliferation and FET expression by comparing FET expression in proliferating and growth arrested cells. However, no relationship between active proliferation and FET protein expression was seen in these experiments. In support of our data is an earlier study showing that FUS expression is uncorrelated to proliferative status [47]. Thus, we conclude differentiation rather than proliferation as an expression determinant for the FET family proteins.

FET proteins have been shown to be part of the splicing machinery [2] and oncogenic variants of the FET proteins are reported to promote aberrant splicing [48,49]. Interestingly, altered subcellular localization of the FUS and EWS associated hnRNPA1 protein results in alternative splicing. In addition, an overall change in subcellular distribution of splicing factors has been proposed to influence pre-mRNA processing [50]. We thus speculate that the heterogeneous tissue and cell type-specific expression patterns shown by the FET proteins and their involvement in RNA processing link these proteins to cell type-specific splicing. The miRNA profile of a given cell could in a similar manner be affected by FET protein expression as many miRNAs are spliced out of introns of protein-coding genes [51]. The tumor type-specific FET fusion oncogenes present in multiple human cancers have documented strong transforming properties and the tumors display few other cytogenetic aberrations [52,53]. As alternative splicing and miRNA maturation are emerging as central for both development and disease [54,55], abnormal FET oncoproteins disturbing these vital processes could thereby instigate significant biological changes resulting in cancer. Analogously, altered adhesion and stress response are common traits of many human cancers and these properties could be targeted by oncogenic variants of FET proteins.

## Conclusion

We conclude that the FUS, EWS and TAF15 proto-oncoproteins are regulated and expressed in a cell type-specific manner. Our results expand on previous knowledge and imply multiple functions for the FET family proteins in specialized cells during both normal and stress-induced situations. These results suggest functions for all FET family members in differentiation, stress response and cell spreading in addition to their previously known activities as transcription factors. The multifunctionality of the FET family proteins makes them vulnerable targets for cancer-causing mutations as such events could affect several cellular control systems simultaneously. A deregulation of multiple vital processes may explain why FET oncogenes resulting from single mutations can disturb cellular homeostasis and in the extension lead to the development of cancer.

## Methods

### Immunohistochemistry

Human normal organ tissue arrays (Super Bio Chips) containing 59 core biopsies per slide were stained with primary antibodies against human FUS [56], EWS and TAF15 (Additional file 4), according to the protocol supplied by the manufacturer. Briefly, slides were deparaffinized in xylene and rehydrated by successive incubations in ethanol followed by immersion in water. Antigens were retrieved by microwave treatment in citrate buffer pH 6.0 and endogenous peroxidase activity was quenched using hydrogen peroxide. Slides were incubated with FET antibodies in Tris-buffered saline pH 7.4 with 2% bovine serum albumin (BSA, Sigma-Aldrich) and 0.05% Tween 20 (Sigma-Aldrich) overnight at 4°C. Primary antibodies were detected using biotin conjugated secondary antibodies (Multi Link, DakoCytomation), which were further incubated with Streptavidin/HRP (P0397, DakoCytomation) and then treated with metal enhanced DAB solution (Pierce). Tissue arrays were counterstained with Mayer's hematoxylin (Histolab), dehydrated in ethanol and xylene, and mounted in Pertex (Histolab). Slides were analyzed with an Olympus BX51 light microscope and selected tissues were photographed on a Nikon Eclipse E1000M light microscope fitted with a ProgRes 3012 digital camera (Kontron Elektronik). FET expression patterns for individual cell types in each tissue were scored based on the amount of positively staining cells (Table 1). Nuclear and cytoplasmic FET expression was estimated by the following criteria: +++ (100-76% positively staining cells), ++ (75-26% positively staining cells), + (25-1% positively staining cells) and - (negative).

### Cell culture

HT-1080 fibrosarcoma cells [57] and F470 primary human fibroblasts [58] were maintained in RPMI1640 medium (Sigma-Aldrich). HeLa cells (a kind gift from Dr. Tommy Nilsson, Department of Medical and Clinical Genetics, Goteborg University) were cultured in Dulbecco's Modified Eagles Medium High Glucose (E15-011, PAA) and U1242MG glioblastoma cells [59] were grown in Basal Medium Eagle with Earle's salts (41010, Gibco). The neuroblastoma cell line SH-SY5Y [60] was maintained in Minimal Essential Medium with Earle's salts. Growth medium was further supplied with 2 mM L-Glutamine, 10% fetal bovine serum (FBS), penicillin (50 U/ml) and streptomycin (50 μg/ml) and cells were cultured at 37°C in 5% CO_2_. Cells were seeded in Lab-Tek flaskettes (Nalge Nunc International) one day prior to immunofluorescence analysis and transfection (see below). Undifferentiated human embryonic stem cell lines SA 121 (Cellartis AB, Gothenburg, Sweden), HUES1 [61] and HUES3 [61] were maintained on mitotically inactivated mouse embryonic fibroblasts (in the Semb laboratory). SA121 was mechanically passaged every 4–7 days and half of medium was changed every second day as previously reported [62]. HUES1 and HUES3 were enzymatically passaged and cultivated as described [61]. In vitro experiments using hESCs were performed according to Swedish ethical guidelines. Stress experiments were performed by treating selected cell types with 0.5 mM sodium arsenite (Sigma-Aldrich) or heat-shock at 44°C for 1 h as described [12].

### Immunofluorescence

Cells were fixed in 3.7% formaldehyde in phosphate-buffed saline (PBS) pH 7.2 at 37°C and stained with primary antibodies for FUS, EWS, TAF15 (Additional file 4) in PBS supplied with 2% BSA and 0.2% Triton x-100 (Merck). Spreading initiation centers were assayed in F470 cells three hours after seeding and in HT1080 cells 16 hours after seeding and stained with antisera directed at Vinculin, FAK and RACK1 (Additional file 4). Stress granules were detected with a TIA-1 antibody (Additional file 4). Primary antibodies were detected using goat Cy3 conjugated secondary antibodies (Fluorolink, Amersham Biosciences) or combinations of donkey/goat/rabbit AlexaFluor 488/568 conjugated secondary antibodies (Molecular Probes). Slides were mounted using Prolong Gold antifade with DAPI (Molecular Probes) and allowed to cure overnight. Cellular fluorescence was imaged using a Zeiss LSM510 META confocal microscope system or a Zeiss Axioplan 2.

### Transfection

The full-length coding regions of *FUS*, *EWSR1 *and *TAF15 *were cloned into EGFP-N1 expression vectors (Clontech) as described [58]. A FUS mutant (here named FUSA) expressing amino acids 1–175 was generated in a similar manner. Cloning primer sequences are available as supplementary data (Additional file 5). FET-EGFP expression vectors were transiently transfected into cells using the FuGENE 6 transfection reagent (Roche), according to the instructions supplied by the manufacturer. On the following day, transfected cells were fixed, mounted and imaged as before. HT-1080 cells stably expressing FUS, FUSA, EWS and TAF15 tagged with EGFP as well as control cells expressing EGFP were obtained after two weeks of prolonged culture of transfected cells under selection of 800 μg/ml geneticin (G418, Invitrogen). These cells were subsequently subjected to single-cell dilution cloning. Stable transfectants were maintained in RPMI1640 medium supplied with 500 μg/ml geneticin. Stable transfectants were fixed and imaged as before. Total protein was obtained by lysis in radioimmunoprecipitation (RIPA) buffer (50 mM Tris-HCl pH 7.4, 150 mM NaCl, 1 mM EDTA, 1% Triton x-100, 1% Sodium deoxycholate, 0.1% SDS) containing 1× Complete Mini Protease Inhibitor (Roche). For cell measurements, stable transfectants were fixed as earlier and stained with 0.2% Evans Blue (Merck) in PBS (whole-cell staining) before red fluorescence was recorded with an Axio Imager Z1 microscope (Zeiss). Images were thresholded for background and average cell areas were obtained by using the "Analyze Particles" function in the public domain ImageJ software.

### Differentiation and proliferation assays

Differentiation of neuroblastoma cells was performed as previously described [63]. Briefly, SHSY-5Y cells were treated with 1 μM all-trans retinoic acid for 3, 6 or 9 consecutive days to induce neuronal differentiation. Treatment with vehicle (DMSO) was used as control. Protein extracts were obtained by lysis in buffer containing 1% Nonidet-P40, 10% glycerol, 20 mM Tris buffer pH 8, and 197 mM NaCl. In addition, FET protein expression was assayed in actively proliferating and quiescent F470 cells as well as in serum stimulated and starved HT-1080 cells. For F470 cells, 80% confluent, actively proliferating cells were lysed and compared with contact-inhibited cells that had grown to full confluence over seven days. For HT-1080 cells, serum-free medium was added to 50% confluent cells grown in 6-well plates for 24 hours after which half of the wells received full-serum medium (10% FBS) and the rest serum-free medium for 20 hours. Total protein was extracted with RIPA lysis buffer as before.

### Western blot

Protein concentrations in cell extracts were determined using a bicinchoninic acid (BCA) protein assay kit (Pierce) and diluted for equal loading on gels. Samples were mixed with 4× LDS sample buffer (Invitrogen), 10% 0.5 M dithiothreitol and run on NuPage 4–12% Bis-Tris gels (Invitrogen). Proteins were blotted onto PVDF membranes (Immobilon) and probed with FET antibodies (Additional file 4). GFP protein was detected using an anti-GFP antibody and beta actin expression was used as loading control (Additional file 4). Bands were visualized by horseradish peroxidase-conjugated secondary antibodies by chemiluminscent detection (SuperSignal West Dura Extended Duration Substrate, Pierce) or alternatively membranes were developed with BCIP/NBT tablets (Sigma-Aldrich). Chemiluminscent membranes were imaged using a FluorChem imaging system (Alpha Innotech Corporation) and bands were quantified using ImageJ.

### Quantitative real-time PCR

Inner and peripheral rings of hESC colonies were separated mechanically and total RNA was extracted with GenElute Mammalian Total RNA kit (Sigma-Aldrich). RNA concentrations were measured with the NanoDrop ND-1000 spectrophotometer. Reverse transcription was performed with SuperScript III (Invitrogen) according to the instructions of the manufacturer using a mixture of 2.5 μM oligo(dT) and 2.5 μM random hexamers (both Invitrogen) as primers. Real-time PCR measurements were performed on an ABI PRISM 7900HT Sequence Detection System (Applied Biosystems). Twenty microliter reactions contained 10 mM Tris (pH 8.3), 50 mM KCl, 3 mM MgCl_2_, 0.3 mM dNTP, 1 U JumpStart Taq polymerase (all Sigma-Aldrich), 0.5 × SYBR Green I (Invitrogen) and 400 nM of each primer (MWG-Biotech). Primer sequences are available as supplementary data (Additional file 5). 1× Reference Dye (Sigma-Aldrich) was used as passive reference dye. Formation of correctly sized PCR products was confirmed by agarose gel electrophoresis (2%) for all assays and melting curve analysis for all samples. Gene expression data was normalized against *ACTB *expression after reference genes evaluation [64].

### Statistical analysis

Spearman's rank correlation coefficients were calculated for nuclear and cytoplasmic expression of FET proteins in tissues using the SPSS 15.0 software. Coefficients were considered significant at the 0.01 level using two-tailed tests.

## Abbreviations

EGFP: enhanced green fluorescent protein; FBS: fetal bovine serum; FET: FUS-EWS-TAF15; hESC: human embryonic stem cells; miRNA: microRNA; PBS: phosphate-buffered saline; RA: all-trans retinoic acid; RIPA: radioimmunoprecipitation; RQ: relative quantification; SIC: spreading initiation center; SG: stress granule; TBS: tris-buffered saline; TFIID: transcription factor II D; TMA: tissue microarray.

## Authors' contributions

MKA and PÅ conceived of the study. MKA, AS, YA and PÅ designed the experiments. MKA, AS, YA and AO performed the experiments. MKA, AS, HS, GS, ON and PÅ analyzed the data. MKA wrote the manuscript with feedback from all authors. All authors read and approved the final manuscript.

## Supplementary Material

Additional file 1 Immunofluorescence and transfections. Immunostaining and transfections of FET proteins. (a) Endogenous and transient FET protein expression in four different cultured cell types with DAPI staining of nuclei (blue). Scale bars indicate 10 μM. (b) Stable expression of FET-EGFP proteins in HT1080 with DAPI staining (blue). Scale bars indicate 10 μM. Click here for file

Additional file 2 Cell area measurements. Clones of stable transfectants were seeded out, stained and imaged as described in Materials and methods. Average cell areas were calculated from 130–200 cells in five images per clone. Error bars show standard error of mean. Click here for file

Additional file 3 Proliferation assay. Western blot showing FET expression in contact-inhibited and actively proliferating F470 cells as well as in serum stimulated and starved HT1080 cells. Beta actin expression is used as a loading control. Click here for file

Additional file 4 Primary antibodies. Primary antibodies used. Click here for file

Additional file 5 Primer sequences. Primers used for cDNA cloning and quantitative real-time PCR. Click here for file
