# Supplementary figures and images for: The multifunctional FUS, EWS and TAF15 proto-oncoproteins show cell type-specific expression patterns and involvement in cell spreading and stress response
Source: BMC Cell Biol. 2008 Jul 11;9:37. doi: 10.1186/1471-2121-9-37 (PMC2478660; doi:10.1186/1471-2121-9-37)

**(a)**

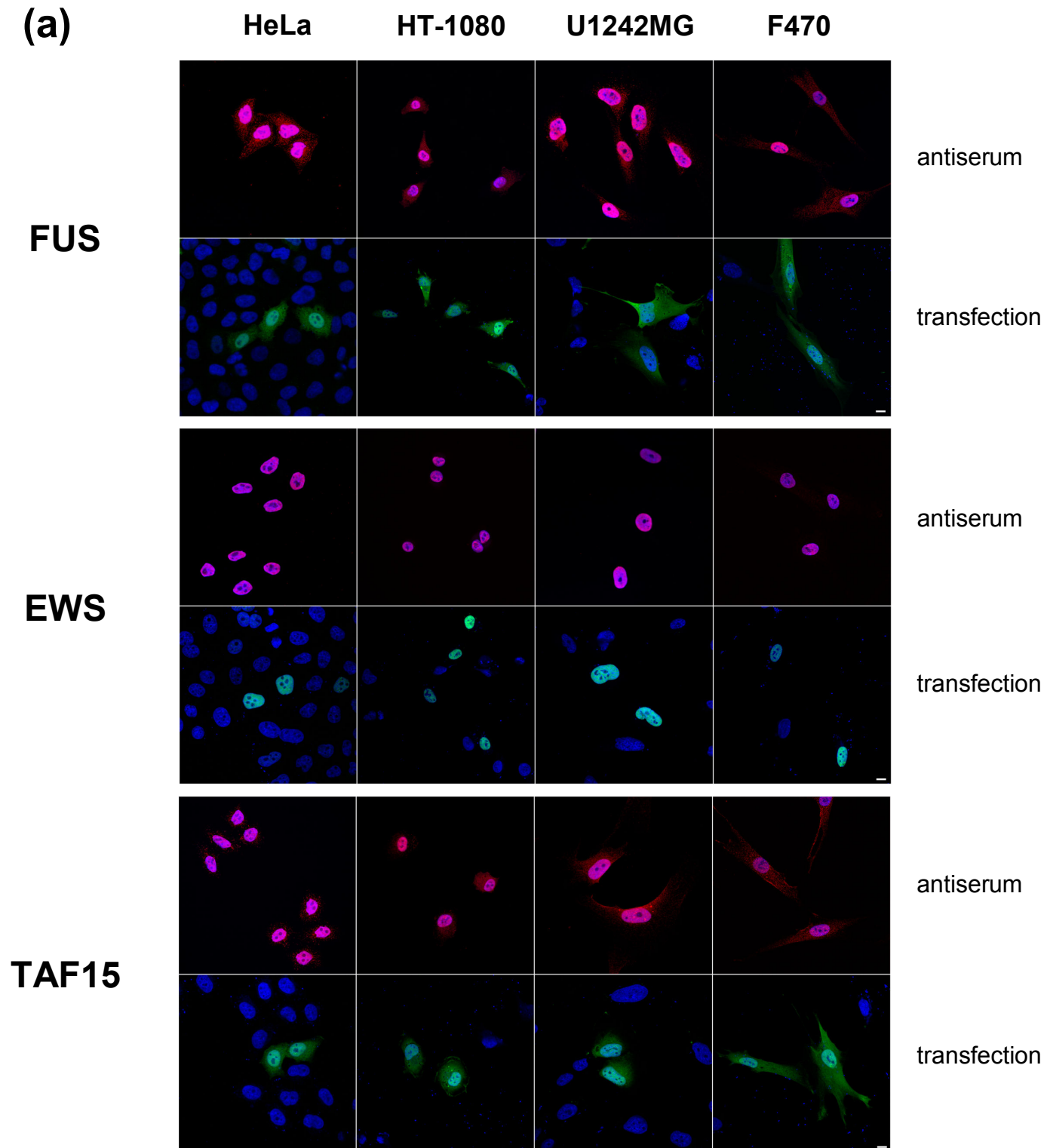

**(b)**

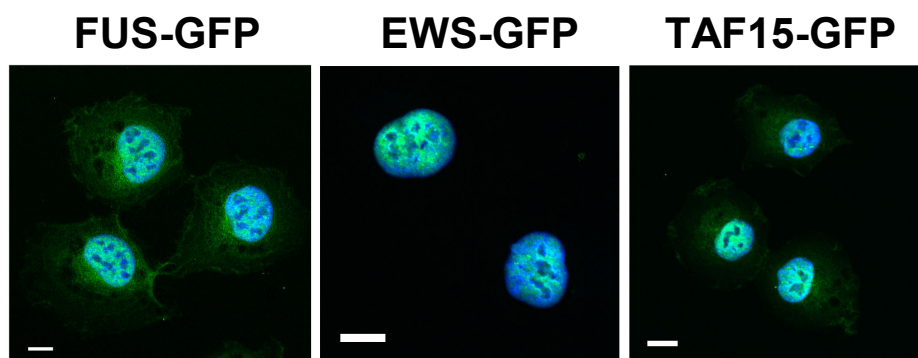

Supplement: Additional file 1 — Immunofluorescence and transfections. Immunostaining and transfections of FET proteins. (a) Endogenous and transient FET protein expression in four different cultured cell types with DAPI staining of nuclei (blue). Scale bars indicate 10 μM. (b) Stable expression of FET-EGFP proteins in HT1080 with DAPI staining (blue). Scale bars indicate 10 μM. [file 1471-2121-9-37-S1.pdf]

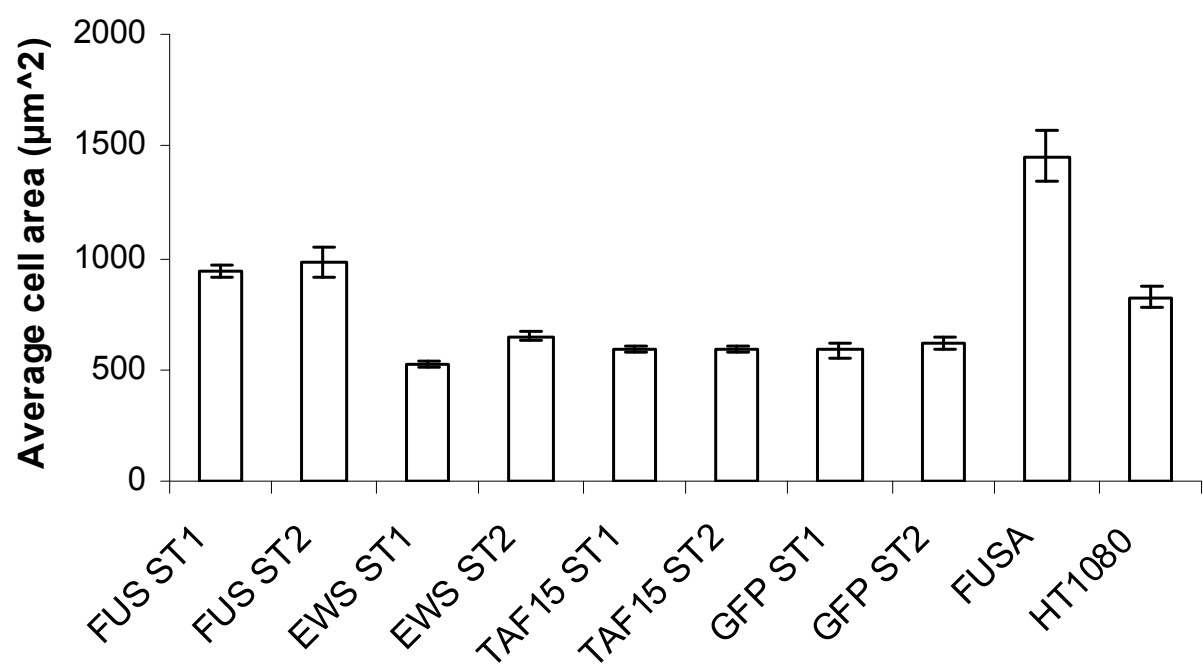

Supplement: Additional file 2 — Cell area measurements. Clones of stable transfectants were seeded out, stained and imaged as described in Materials and methods. Average cell areas were calculated from 130–200 cells in five images per clone. Error bars show standard error of mean. [file 1471-2121-9-37-S2.pdf]

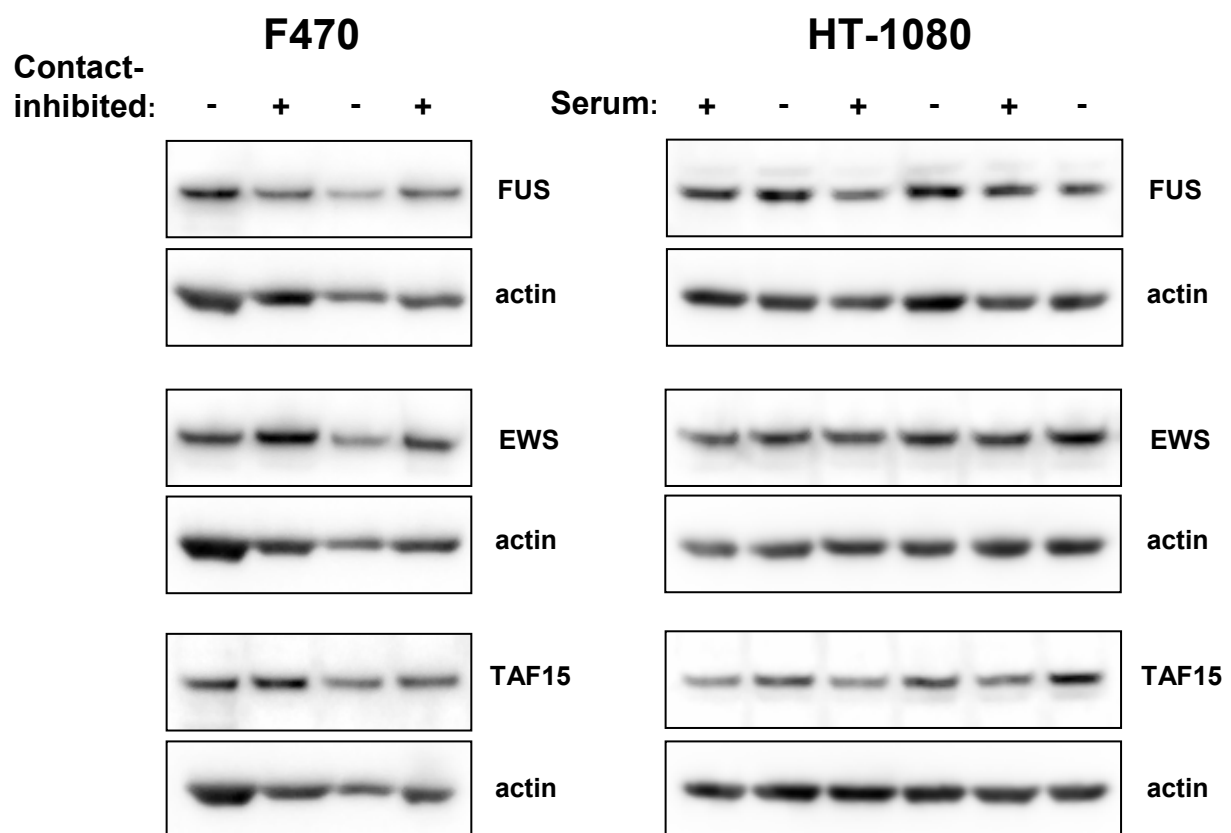

Supplement: Additional file 3 — Proliferation assay. Western blot showing FET expression in contact-inhibited and actively proliferating F470 cells as well as in serum stimulated and starved HT1080 cells. Beta actin expression is used as a loading control. [file 1471-2121-9-37-S3.pdf]
