# Supplementary material for: The multifunctional FUS, EWS and TAF15 proto-oncoproteins show cell type-specific expression patterns and involvement in cell spreading and stress response
Source: BMC Cell Biol. 2008 Jul 11;9:37. doi: 10.1186/1471-2121-9-37 (PMC2478660; doi:10.1186/1471-2121-9-37)
Supplement: Additional file 4 — Primary antibodies. Primary antibodies used. [file 1471-2121-9-37-S4.doc]

Supplementary table 1 – Primary antibodies

| Antigen | Species | Clonality | Manufacturer | Catalogue no. | Clone | IHC | IF | WB |
| --- | --- | --- | --- | --- | --- | --- | --- | --- |
|  |  |  |  |  |  |  |  |  |
| beta Actin | M | mAb | Abcam | mAbcam 8226 | - |  |  | X |
| EWS | M | mAb | Santa Cruz Biotechnologies | sc-28327 | G-5 | X | X | X |
| FAK | G | pAb | Santa Cruz Biotechnologies | sc-557-G | A-17 |  | X |  |
| FUS | R | pAb | not commercially available | - | - | X | X | X |
| FUS/TLS | M | mAb | Santa Cruz Biotechnologies | sc-47711 | 4H11 |  |  | X |
| GFP | M | mAb | Clontech | 632381 | JL-8 |  |  | X |
| RACK1 | R | pAb | Santa Cruz Biotechnologies | sc-10775 | H-187 |  | X |  |
| TAF15 | R | pAb | Genetex Inc. | GTX77901 | - | X | X |  |
| TAFII68 | M | mAb | Millipore | MAB3672 | - |  | X | X |
| TIA-1 | G | pAb | Santa Cruz Biotechnologies | Sc-1751 | c-20 |  | X |  |
| Vinculin | M | mAb | Sigma-Aldrich | V9131 | hVIN-1 |  | X |  |

Abbreviations: IF – used for immunofluorescence, IHC – used for immunohistochemistry, G – goat, M – mouse, R- rabbit, mAb – monoclonal antibody, pAb – polyclonal antibody, WB – used for western blot
