# Supplementary material for: The multifunctional FUS, EWS and TAF15 proto-oncoproteins show cell type-specific expression patterns and involvement in cell spreading and stress response
Source: BMC Cell Biol. 2008 Jul 11;9:37. doi: 10.1186/1471-2121-9-37 (PMC2478660; doi:10.1186/1471-2121-9-37)
Supplement: Additional file 5 — Primer sequences. Primers used for cDNA cloning and quantitative real-time PCR. [file 1471-2121-9-37-S5.doc]

### cDNA cloning primers

| FUS_F_EcoRI | GTTGGAATTCGTTGCTTGCTT |
| --- | --- |
| FUS_R_BamHI | TATGGATCCATACGGCCTCTCCCTGCGATCCTG |
| FUSA_F_XhoI | TATACTCGAGCGCGGACATGGCCTCAAACG |
| FUSA_R_BamHI | TATTGGATCCACTCCACCTCCACCTC |
| EWS_F_XhoI | ATACTCGAGATGGCGTCCACGGATTACAG |
| EWS_R_BamHI | TTAGGATCCGCGTAGGGCCGATCTCTGCG |
| TAF15_F_XhoI | ATACTCGAGATGTCGGATTCTGGAAGTTA |
| TAF15_R_BamHI | TATGGATCCGCGTATGGTCGGTTGCGCTGA |

### Quantitative real-time PCR primers

| FUS fwd | AAACAAGAAAACGGGACAGC |
| --- | --- |
| FUS rew | GGCGAGTAGCAAATGAGACC |
| EWS fwd | TACCCACCCCAAACTGGAT |
| EWS rew | GACTCCTGCCCATAAACACC |
| TAF15 fwd | GTGGCTATGGAGGCAAAATG |
| TAF15 rew | GAAAGCAGCAGGCAAAACTC |
| SOX2 fwd | TACCTCTTCCTCCCACTCCA |
| SOX2 rew | CCCATTTCCCTCGTTTTTCT |
| CCNA2 fwd | GCTGCCTTTCATTTAGCACTC |
| CCNA2 rew | CTGGTGGGTTGAGGAGAGAA |
| VIM fwd | AGATTCCACTTTGCGTTCA |
| VIM rew | ACCGCTTCGCCAACTACAT |
| POU5F1 fwd | CGAAAGAGAAAGCGAACCAG |
| POU5F1 rew | AACCACACTCGGACCACATC |
